# Anti-MDA5 antibody as a potential diagnostic and prognostic biomarker in patients with dermatomyositis

**DOI:** 10.18632/oncotarget.15716

**Published:** 2017-02-24

**Authors:** Liubing Li, Qian Wang, Funing Yang, Chanyuan Wu, Si Chen, Xiaoting Wen, Chenxi Liu, Yongzhe Li

**Affiliations:** ^1^ Department of Rheumatology and Clinical Immunology, Peking Union Medical College Hospital, Chinese Academy of Medical Sciences & Peking Union Medical College, Key Laboratory of Rheumatology and Clinical Immunology, Ministry of Education, Beijing, China; ^2^ Department of Medical Laboratory, The First Hospital of Jilin University, Changchun, China; ^3^ Department of Clinical Laboratory, Beijing Anzhen Hospital, Capital Medical University, Beijing, China

**Keywords:** dermatomyositis, anti-MDA5, diagnosis, prognosis, marker

## Abstract

The presence of anti-MDA5 antibodies in serum represents an important biomarker in the diagnosis and prediction of prognosis for patients with idiopathic inflammatory myopathies (IIMs). Due to conflicting results that have been reported regarding the detection of anti-MDA5 antibodies, the goal of this study was to assess a potential association between the presence of anti-MDA5 antibodies and dermatomyositis/polymyositis (DM/PM), as well as the diagnostic and prognostic values of anti-MDA5 antibodies for DM/PM. For this, a review of literature published prior to October 15, 2016 was conducted. Eight studies with 286 PM patients and 216 healthy controls and nine studies with 628 DM patients and 221 healthy controls were selected according to specific inclusion criteria. The outcomes of these studies revealed that the presence of anti-MDA5 antibodies was associated with DM, especially CADM, and not with PM. Furthermore, the pooled sensitivity, specificity, and area under the curve (AUC) values were 0.62 (95% confidence interval (CI): 0.52–0.70), 1.00 (95% CI: 0.97–1.00), and 0.9381 for CADM patients versus healthy controls when an immunoprecipitation method was used. The presence of anti-MDA5 antibodies was also found to be significantly associated with an increased risk of death in DM (relative risk = 3.32, 95% CI: 1.65–6.67, *P* = 0.001). These findings suggest that anti-MDA5 antibodies correlate with DM and could be used as a biomarker in the clinical diagnosis of CADM. The presence of anti-MDA5 antibodies was also associated with poor prognosis regarding the overall survival of patients with DM.

## INTRODUCTION

Idiopathic inflammatory myopathies (IIMs) are rare autoimmune diseases with an annual incidence of 5.8 to 7.9 cases per 100000, and an annual prevalence of 14.0 to 17.4 patients per 100000 in the United States [1]. Dermatomyositis (DM) and polymyositis (PM) are the two main subtypes of IIMs, and these conditions are characterized by proximal muscle weakness and inflammation on muscle biopsies [2]. Additionally, classic DM and clinically amyopathic dermatomyositis (CADM) are two classifications of DM according to skin and/or muscle involvement at presentation. For patients with IIMs that also have interstitial lung disease (ILD), they have a higher morbidity and mortality than patients without ILD [3, 4]. Therefore, it is important to diagnose IIMs in their early stages and to accurately evaluate prognosis upon presentation.

Electromyography and muscle biopsies are often used to diagnose IIMs. However, these methods are invasive. In contrast, myositis-specific antibodies (MSAs) have been found to be useful for obtaining a diagnosis and for predicting the prognosis of IIMs [5]. In addition, tests with MSAs are painless and convenient. In recent years, a number of MSAs have been identified in patients with IIMs, including those that recognize transcription intermediary factor 1 gamma (TIF1γ), Mi-2, small ubiquitin-like modifier activating enzyme (SAE), and melanoma differentiation-associated gene5 (MDA5). The latter, also known as IFN induced with helicase C domain protein 1 (IFIH1), is a cytoplasmic sensor of viral nucleic acids that regulates innate immune responses [7, 8]. The MDA5 antibody, also known as an anti-CADM-140 antibody, has especially been associated with CADM [6], and has been used to detect dermatopulmonary syndrome in patients who have undergone allogeneic hematopoietic stem cell transplantation [9] or in patients with DM [10, 11].

However, results regarding an association between the presence of anti-MDA5 antibodies and IIMs, as well as the diagnostic accuracy of this antibody for IIMs, have been inconsistent. Sato et al. reported that anti-MDA5 antibodies were associated with IIMs [6], while Bodoki et al. reported that none of the patients with IIMs that they examined were positive for anti-MDA5 antibodies [12]. Therefore, we conducted a meta-analysis to analyze published data regarding an association between anti-MDA5 antibodies and DM/PM, and to assess the diagnostic value and prognostic significance of anti-MDA5 antibodies for these diseases.

## RESULTS

### Characteristics of the examined studies

**Figure 1:**
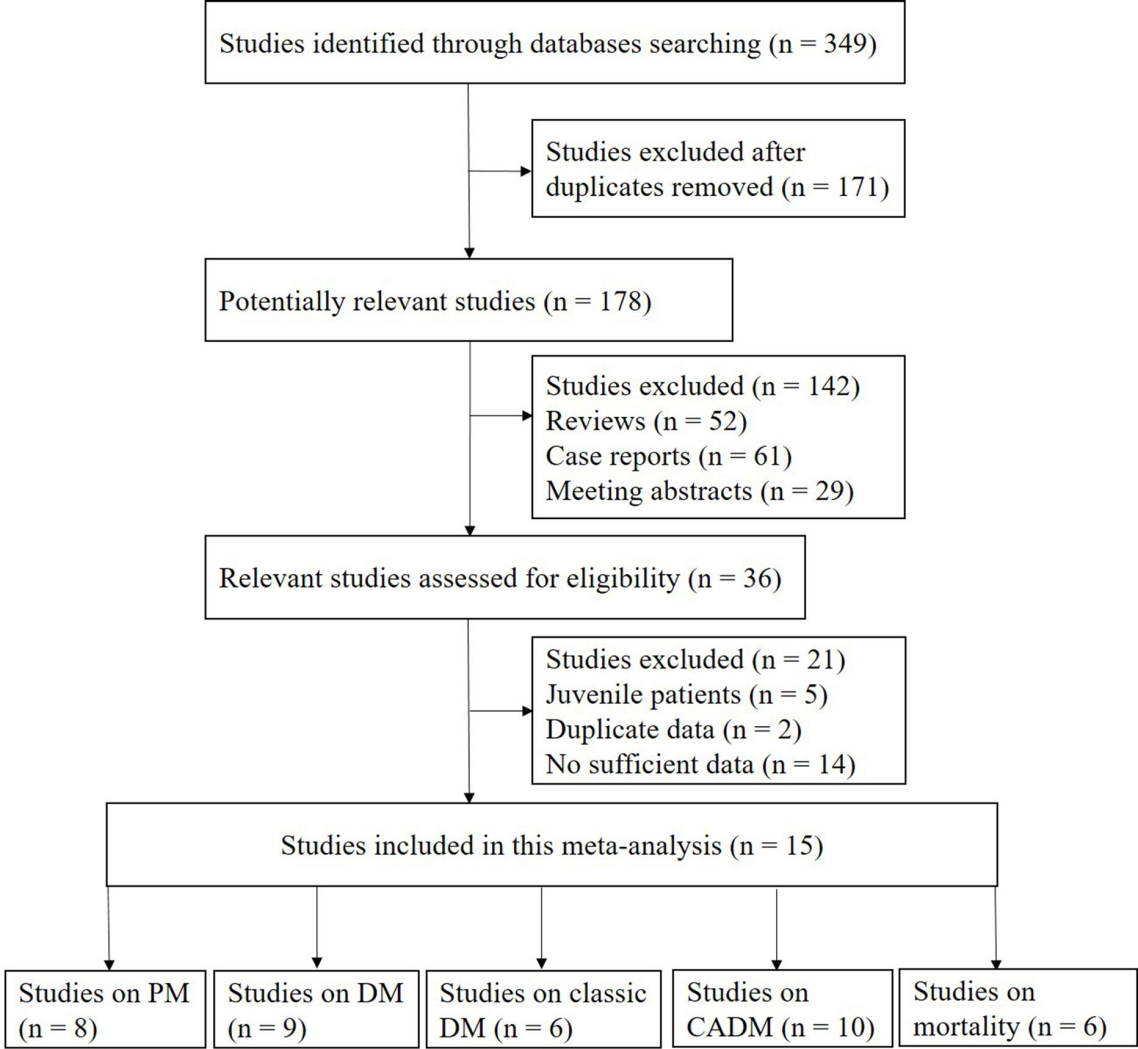
Flow chart of the study selection procedure used

A total of 349 potentially relevant articles were identified from systematic searches performed of the PubMed, EMBASE, Web of Science, Cochrane Library, and Scopus databases (see Materials and methods). As shown in Figure 1, fifteen eligible studies were selected for analysis. Of these studies, eight involved cases of PM [6, 8, 10, 11, 13–16], nine involved cases of DM [6, 8, 10, 11, 13–15, 17, 18], six involved cases of classic DM [6, 8, 11, 14–16], ten involved cases of CADM [6, 8, 10, 11, 14–16, 18–20], and six involved mortality [8, 10, 14, 21–23]. The characteristics of each eligible study are summarized in Supplementary Tables 1 and 2.

### Associations between anti-MDA5 antibodies and DM/classic DM/CADM/PM

**Figure 2:**
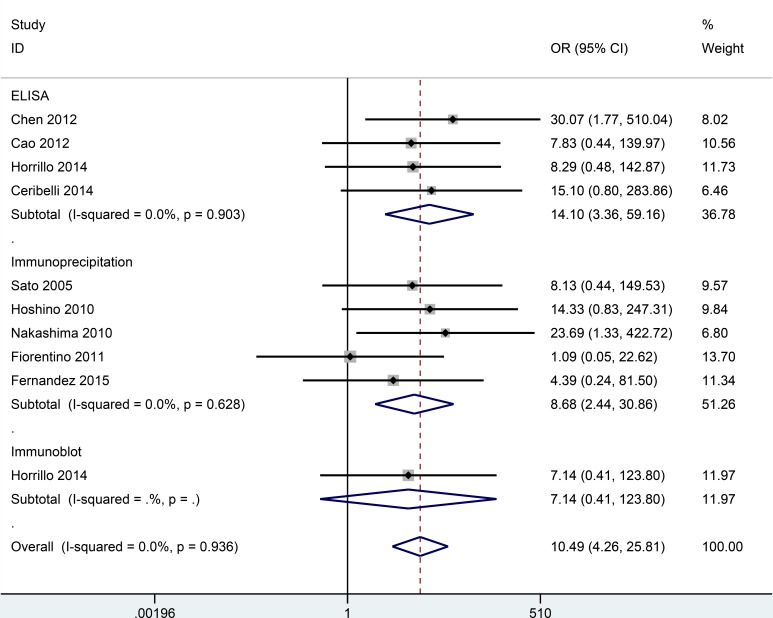
Forest plot of the association between the presence of anti-MDA5 antibodies and DM

**Figure 3:**
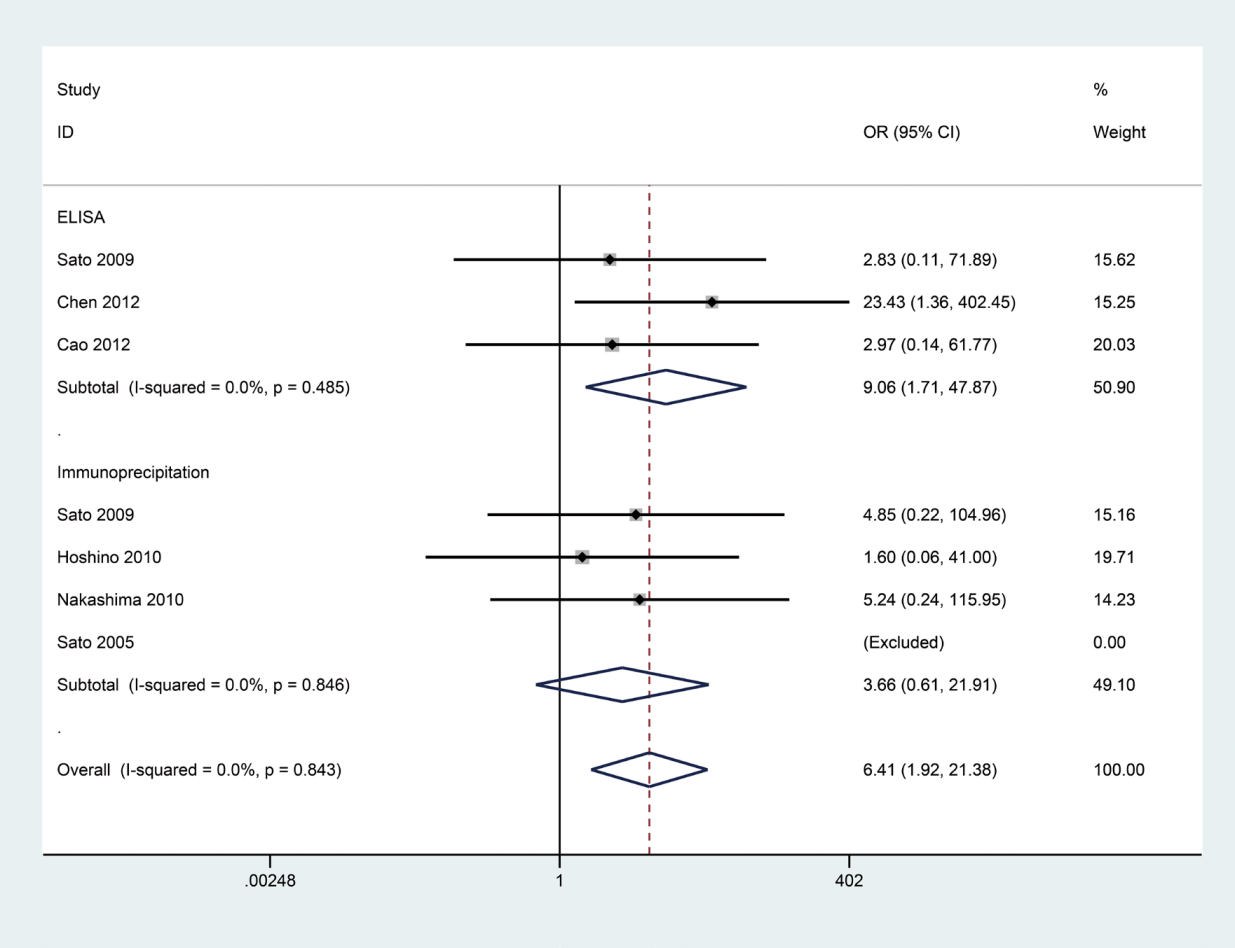
Forest plot of the association between the presence of anti-MDA5 antibodies and classic DM

**Figure 4:**
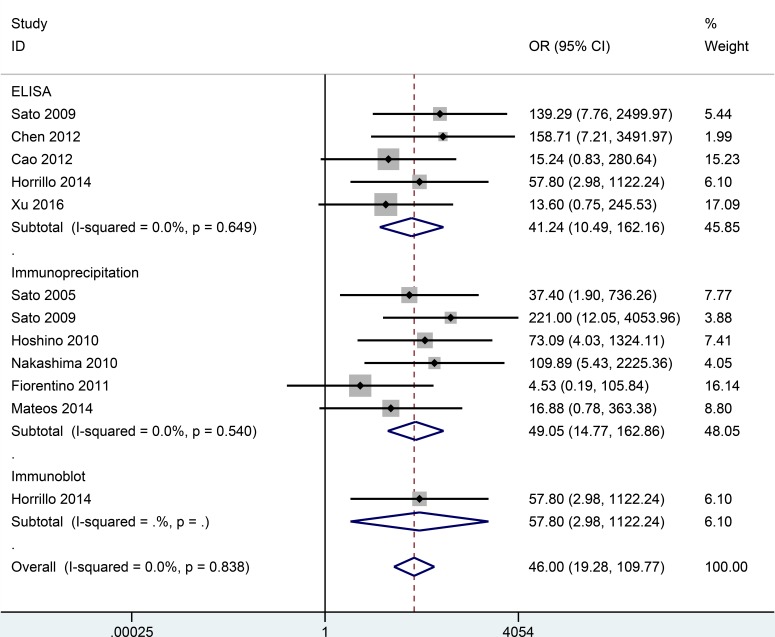
Forest plot of the association between the presence of anti-MDA5 antibodies and CADM

No substantial heterogeneity was observed by using a fixed-effects model to calculate the pooled odds ratio (OR) (*P* > 0.10 and *I^2^* < 50%). Correlation data between anti-MDA5 antibodies and DM/classic DM/CADM are listed in Figures 2, 3, 4. However, no association between anti-MDA5 antibodies and PM was observed (Supplementary Figure 1, OR = 2.93, 95% confidence interval (CI): 0.14–63.49, *P* = 0.493).

### Associations between anti-MDA5 antibodies and DM risk

The overall OR showed that the frequency of anti-MDA5 antibodies in patients with DM was significantly higher than in healthy controls (OR = 10.49, 95% CI: 4.26–25.81, *P* < 0.001) (Figure 2). This finding was based on an analysis of nine studies involving 628 DM patients and 221 healthy controls. When a stratified analysis was conducted according to detection method, a significant increase in DM risk was associated with the detection of anti-MDA5 antibodies by enzyme-linked immunosorbent assay (ELISA) (OR = 14.10, 95% CI: 3.36–59.16, *P* < 0.001) and immunoprecipitation (OR = 8.68, 95% CI: 2.44–30.86, *P* = 0.001). Moreover, in a study of 117 DM patients versus 25 healthy controls that employed an immunoblot method, anti-MDA5 antibodies did not correlate with DM (OR = 7.14, 95% CI: 0.41–123.80, *P* = 0.177). However, the latter results should be interpreted with caution due to the small sample size examined.

### Associations between anti-MDA5 antibodies and classic DM risk

The frequency of anti-MDA5 antibodies in patients with classic DM was significantly higher than in the healthy controls (OR = 6.41, 95% CI: 1.92–21.38, *P* = 0.003) (Figure 3). In the current study of DM cases, three studies of 143 classic DM patients versus 94 healthy controls using ELISA and four studies of 123 classic DM patients versus 89 healthy controls using immunoprecipitation were examined. In a stratified analysis according to detection method, anti-MDA5 antibodies were associated with classic DM with the ELISA method (OR = 9.06, 95% CI: 1.71–47.87, *P* = 0.010), yet an association was not observed when an immunoprecipitation method was used (OR = 3.66, 95% CI: 0.61–21.91, *P* = 0.155).

### Associations between anti-MDA5 antibodies and CADM risk

The frequency of anti-MDA5 antibodies was significantly higher in patients with CADM than in healthy controls. For example, the pooled OR from ten studies involving 212 CADM patients and 214 healthy controls was 46.00 (95% CI: 19.28–109.77, *P* < 0.001) (Figure 4), and this value was notably higher than that for patients with DM/classic DM versus healthy controls. Additionally, in the stratified analysis performed according to detection method, anti-MDA5 antibodies were significantly associated with CADM risk when: ELISAs were used to evaluate samples from 127 CADM patients and 134 healthy controls (OR = 41.24, 95% CI: 10.49–162.16, *P* < 0.001), in immunoprecipitation assays that compared 117 CADM patients and 112 healthy controls (OR = 49.05, 95% CI: 14.77–162.86, *P* < 0.001), and in immunoblot assays that compared 15 CADM patients and 25 healthy controls (OR = 57.80, 95% CI: 2.98–1122.24, *P* = 0.007). However, additional studies are needed to confirm the association between anti-MDA5 antibodies and CADM risk that was observed with the immunoblot method due to the small sample size that was examined.

### Assessment of threshold effects and heterogeneity

**Figure 5:**
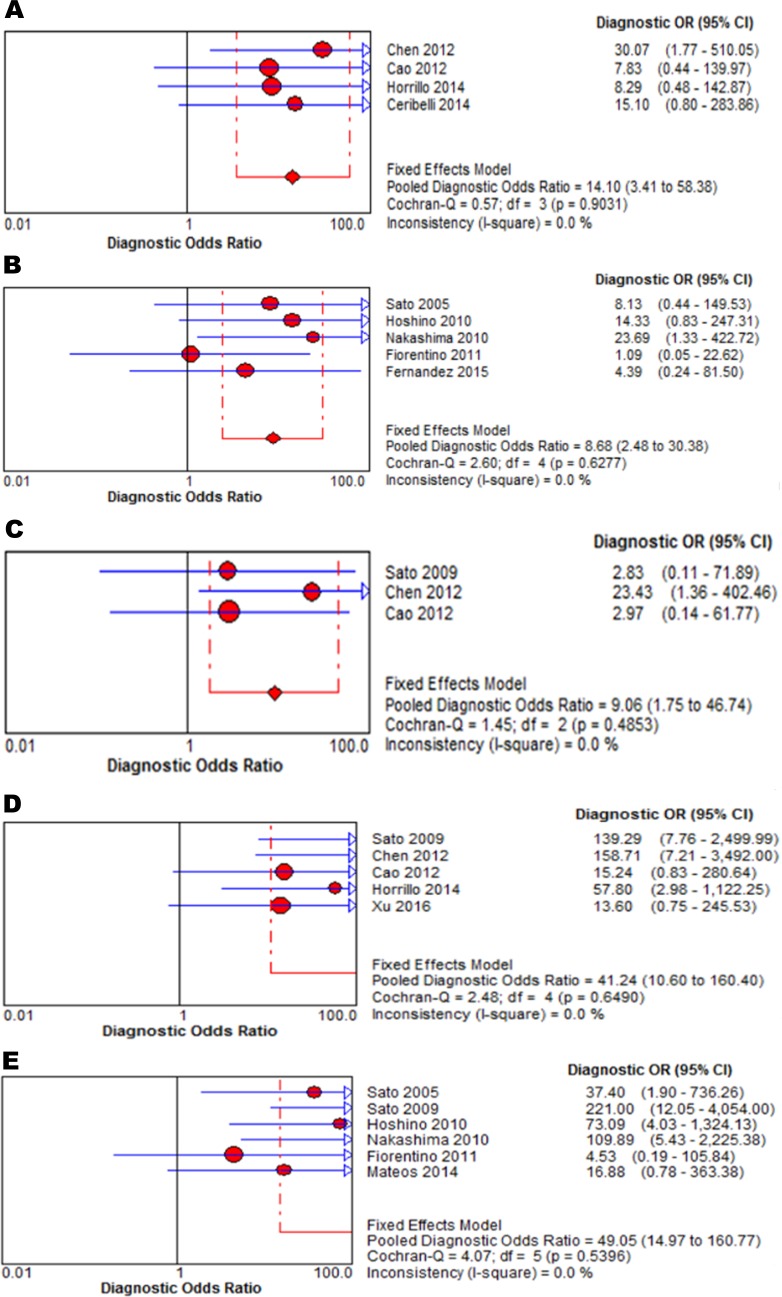
Forest plots of the pooled estimates of DOR of anti-MDA5 antibodies detected by: ELISA in DM patients (**A**), by immunoprecipitation in DM (**B**), by ELISA in classic DM (**C**), by ELISA in CADM (**D**), and by immunoprecipitation in CADM (**E**).

No threshold effects (all *P* > 0.05), nor significant heterogeneity (all *P* > 0.10 and *I^2^* < 50%), were observed (Figure 5). Therefore, a fixed-effects model was used to combine the accuracy indexes, including the pooled sensitivity, pooled specificity, and area under the curve of the summary receiver operating characteristic (AUC) values. The resulting data are summarized in Table 1.

**Table 1:** Diagnostic accuracy of anti-MDA5 antibodies for DM/classic DM/CADM in a stratified analysis according to detection method

Disease type	Method	Pooled sensitivity(95% CI)	Pooled specificity(95% CI)	AUC
DM	ELISA	0.18 (0.14–0.23)	1.00 (0.97–1.00)	0.8589
DM	Immunoprecipitation	0.17 (0.13–0.22)	1.00 (0.96–1.00)	0.8121
Classic DM	ELISA	0.13 (0.08–0.19)	1.00 (0.96–1.00)	0.8167
CADM	ELISA	0.46 (0.38–0.56)	1.00 (0.97–1.00)	0.9301
CADM	Immunoprecipitation	0.62 (0.52–0.70)	1.00 (0.97–1.00)	0.9381

Abbreviations: DM = dermatomyositis; CADM = clinically amyopathic dermatomyositis; ELISA = enzyme-linked immunosorbent assay; CI = confidence interval; AUC = area under the curve of the summary receiver operating characteristic.

### Diagnostic capacity of anti-MDA5 antibodies in patients with DM

A stratification analysis was performed based on the testing methods used. When an ELISA was used to detect anti-MDA5 antibodies, the pooled sensitivity, specificity, and AUC values were 0.18 (95% CI: 0.14–0.23), 1.00 (95% CI: 0.97–1.00), and 0.8589, respectively (Supplementary Figure 2). When immunoprecipitation was used to detect anti-MDA5 antibodies, the pooled sensitivity, specificity, and AUC values were 0.17 (95% CI: 0.13–0.22), 1.00 (95% CI: 0.96–1.00), and 0.8121, respectively (Supplementary Figure 3). Both sets of results suggest that detection of anti-MDA5 antibodies provides a low diagnostic accuracy for DM.

### Diagnostic capacity of anti-MDA5 antibodies for classic DM

The overall sensitivity, specificity, and AUC values for anti-MDA5 antibodies in patients with classic DM were 0.13 (95% CI: 0.08–0.19), 1.00 (95% CI: 0.96–1.00), and 0.8167, respectively (Supplementary Figure 4). The outcomes for these cases demonstrate that the detection of anti-MDA5 antibodies by ELISA had a low diagnostic accuracy for classic DM.

### Diagnostic capacity of anti-MDA5 antibodies for CADM

**Figure 6:**
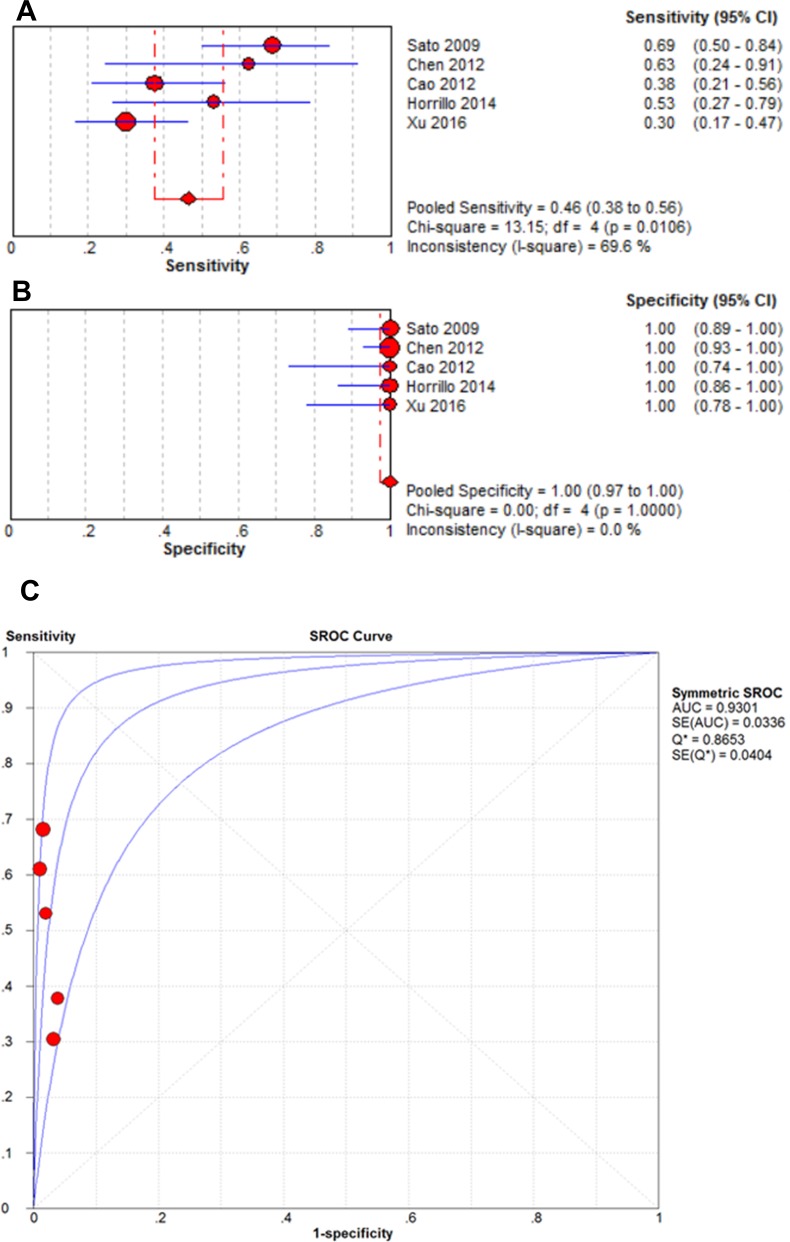
Forest plots of the pooled estimates of sensitivity (**A**), specificity (**B**), and AUC (**C**) values of anti-MDA5 antibodies detected by ELISA in CADM patients.

**Figure 7:**
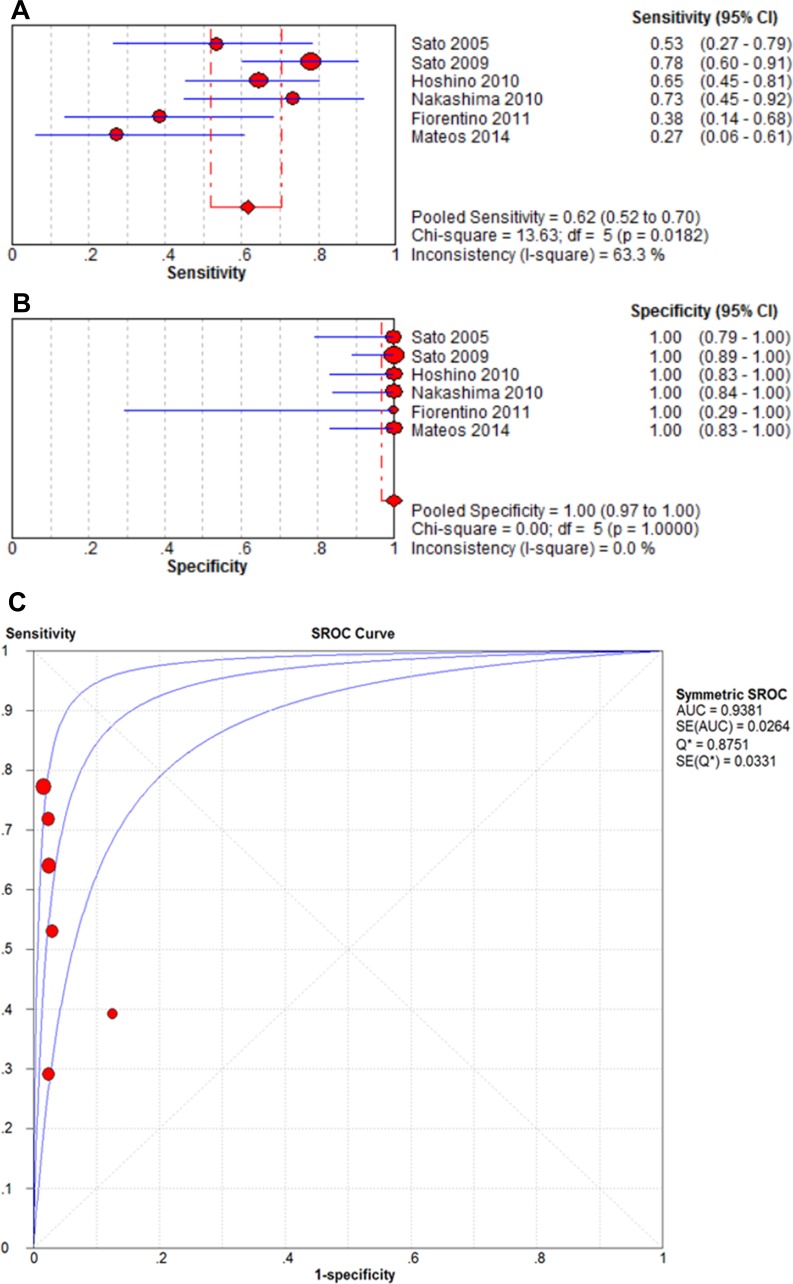
Forest plots of the pooled estimates of sensitivity (**A**), specificity (**B**), and AUC (**C**) values of anti-MDA5 antibodies detected by immunoprecipitation in CADM patients.

The pooled sensitivities when anti-MDA5 antibodies were detected with ELISAs and immunoprecipitation assays in patients with CADM were 0.46 (95% CI: 0.38–0.56) and 0.62 (95% CI: 0.52–0.70), respectively. In contrast, the pooled specificities for these two methods were both 1.00 (95% CI: 0.97–1.00). The AUC values for the two methods were also similar (0.9301 vs. 0.9381, respectively). As shown in Figures 6 and 7, the presence of anti-MDA5 antibodies was associated with a higher diagnostic value for CADM compared with DM/classic DM.

### Prognostic role of anti-MDA5 antibodies for DM patients

**Figure 8:**
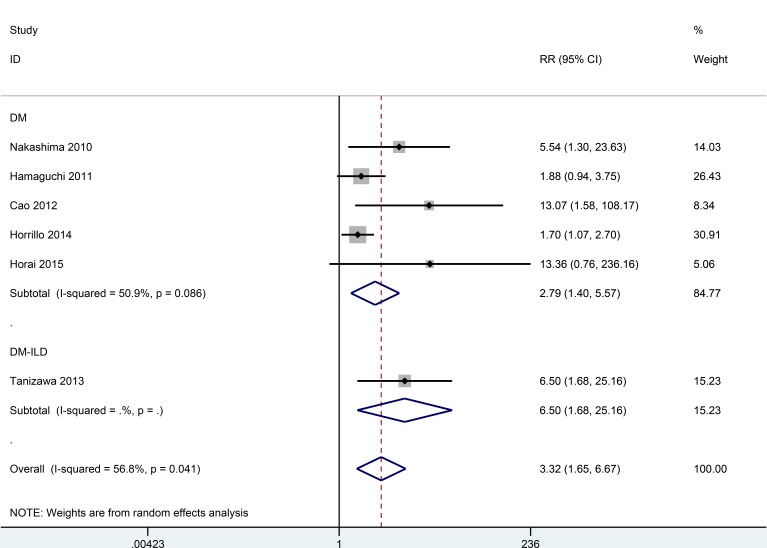
Pooled estimate of RR of DM associated with the presence of anti-MDA5 antibodies

The overall relative risk (RR) determined from six studies with 365 patients with DM was 3.32 (95% CI: 1.65–6.67) (Figure 8). This result suggests that anti-MDA5 antibodies are associated with poor prognosis in regard to the overall survival of DM patients. Furthermore, when a subgroup analysis according to disease type was performed, the pooled RR from a study with 40 DM patients with ILD was 6.50 (95% CI: 1.68–25.16). Therefore, the detection of anti-MDA5 antibodies may provide a poorer prognosis in cases of DM with ILD than in cases of DM without ILD. However, these results should be interpreted with caution due to the small number of cases examined.

## DISCUSSION

It is difficult to acquire a large sample size of DM/PM cases due to the rarity of these two autoimmune diseases. However, it is apparent that early diagnosis and aggressive management are two factors that significantly contribute to control of disease development for both conditions [24]. To assess disease risk, clinicians currently use manifestations and MSAs [25, 26]. MSAs have been found to correlate with clinical features and have also served as diagnostic and prognostic markers. Moreover, for a diagnosis of DM/PM, combination testing of MSAs is necessary. Previously, the presence of anti-TIF1γ antibodies was found to be significantly associated with cancer-associated myositis [27]. For example, in a meta-analysis performed by Ernesto et al. [27], the pooled sensitivity of anti-TIF1γ antibodies was 78%, while the specificity was 89%, in diagnosing cancer-associated DM. The presence of anti-Mi-2 antibodies has also been identified as a favorable prognostic marker and they indicate a good response to immunosuppressive therapy [28]. Meanwhile, anti-SAE antibodies have been associated with cutaneous involvement and a DM phenotype [29]. Anti-MDA5 antibodies have been identified as important MSAs as well. However, in some studies [8, 15], an association between the presence of anti-MDA5 antibodies and DM has been observed, while in other studies [17, 18] it has not. As a result, the diagnostic accuracy and prognostic value of anti-MDA5 antibodies for cases of DM/PM remain confusing. Consequently, the goal of the present meta-analysis was to test the value of detecting anti-MDA5 antibodies in the sera of DM/PM patients.

In the current study, anti-MDA5 antibodies were absent in the sera of the PM patients examined. In contrast, the pooled OR from the comparison of DM, classic DM, CADM, PM, and healthy controls showed that the presence of anti-MDA5 antibodies was significantly associated with CADM. We also conducted a stratification analysis based on the detection method used. Both ELISA and immunoprecipitation methods provided detection of anti-MDA5 antibodies that were relevant to cases of DM. However, only when the ELISA method was used was an association between anti-MDA5 antibodies and classic DM observed. For cases of CADM, a positive correlation was observed between anti-MDA5 antibodies detected with immunoprecipitation, ELISA, and immunoblot assays. However, the latter results should be interpreted with caution due to the small sample size examined. Therefore, additional studies employing the immunoblot method are needed to confirm the observed association between anti-MDA5 antibodies and CADM.

It has been suggested that the presence of anti-MDA5 antibodies may represent a marker for DM [6, 8]. Hence, we further evaluated the diagnostic role of anti-MDA5 antibodies in patients with DM versus healthy subjects. When ELISAs were used to detect anti-MDA5 antibodies in cases of DM, classic DM, and CADM, high specificity (all specificity = 1.00) and low sensitivity (0.18 vs. 0.13 vs. 0.46, respectively) were observed, thereby suggesting that the presence of anti-MDA5 antibodies may not be an appropriate screening index for these diseases. Similarly, when an immunoprecipitation method was used for the detection ofanti-MDA5 antibodies, high specificity (each 1.00) and relatively low sensitivity (0.17 vs. 0.62, respectively) were observed for both DM and CADM. The AUC values also demonstrated that anti-MDA5 antibodies were associated with a higher diagnostic value for CADM than for DM (0.9381 vs. 0.8121, respectively). Taken together, these results suggest that anti-MDA5 antibodies may represent an effective biomarker for CADM.

In some studies, the presence of anti-MDA5 antibodies has correlated with the prognosis of DM [14, 19]. However, in other studies, this correlation was not observed [21, 23]. Here, the prognostic role of anti-MDA5 antibodies was analyzed for 115 patients that carried anti-MDA5 antibodies and for 250 patients that were negative for anti-MDA5 antibodies. We found that the presence of anti-MDA5 antibodies may be associated with poor prognosis in patients with DM. Furthermore, in a stratified analysis according to disease classification, a greater association between the presence of anti-MDA5 antibodies and poor prognosis in overall survival was observed for DM patients with ILD (RR = 6.50) than for DM patients without ILD. However, due to the small number of cases that were examined, additional studies are needed to verify these results.

There were limitations associated with our meta-analysis. First, because we only searched articles published in PubMed, EMBASE, Web of Science, the Cochrane Library, and Scopus, relevant publications in other databases were not evaluated for inclusion. Second, we did not include meeting abstracts due to the limited amount of data they present. Studies from African populations were also limited. Finally, due to the rarity of DM/PM cases, the sample size included in our current study was relatively small, and thus, additional studies are needed to confirm the present results.

In conclusion, detection of anti-MDA5 antibodies was found to correlate with DM, and especially CADM, in the meta-analysis we performed. Moreover, anti-MDA5 antibodies showed good value in diagnosing CADM and were associated with an unfavorable prognosis in DM patients. Thus, well-designed prospective studies with larger sample sizes are warranted to verify the present results.

## MATERIALS AND METHODS

### Literature search strategy

Systematic searches of PubMed, EMBASE, Web of Science, the Cochrane Library, and Scopus were conducted, without language restrictions, to identify studies published by October 15, 2016 that contained the following terms: “MDA5”, “CADM-140”, “melanoma differentiation-associated gene 5”, “polymyositis”, “dermatomyositis”, and “clinically amyopathic dermatomyositis”. References of the retrieved studies and reviews were also manually examined for additional relevant studies.

### Inclusion and exclusion criteria

Studies meeting the following criteria were included: (1) studies with patients other than juveniles that were diagnosed with DM/PM according to criteria proposed by Bohan and Peter [30] or patients with CADM based on criteria suggested by Sontheimer [31] or Sato and Kuwana [32]; (2) studies with healthy donors as controls; and (3) studies that provided sufficient data to evaluate the utility of an anti-MDA5 antibody in the diagnosis of DM/classic DM/CADM/PM. If the same data were published in different articles that met the inclusion criteria, only the study with the largest sample size was included. Reviews, case reports, and meeting abstracts were excluded due to their limited presentation of data.

### Data extraction and quality assessment

Two investigators independently browsed the full text of potentially eligible articles and extracted the relevant data from each study. The following information were collected: first author's name, year of publication, disease type, country of study, ethnicity of the patients examined, detection method, cut-off value, sample type, total number of cases, total number of healthy controls, and frequency and mortality associated with the presence or absence of anti-MDA5 antibodies. Disagreements were resolved by discussion.

### Statistical analysis

Pooled OR with 95% CI were calculated to evaluate the association between anti-MDA5 antibodies and disease, and pooled RR with 95% CI were calculated to evaluate the prognostic value using Stata 12.0 software (Stata Corporation, College Station, TX, USA). Meta-DiSc statistical software (version 1.4, Unit of Clinical Biostatistics, Ramony Cajal Hospital, Madrid, Spain) was used to assess threshold effects, heterogeneity, and to calculate the pooled diagnostic odds ratio (DOR), sensitivity, specificity, and AUC values [33]. When the *P*-value was greater than 0.10 in Q-statistic, or *I^2^* was less than 50% in I^2^-statistic, a fixed-effects model was used to pool the accuracy indexes; otherwise, a random-effects model was used [34].

## SUPPLEMENTARY MATERIALS FIGURES AND TABLES







## Abbreviations

IIMs: idiopathic inflammatory myopathies
DM: dermatomyositis
PM: polymyositis
CADM: clinically amyopathic dermatomyositis
ILD: interstitial lung disease
MSAs: myositis-specific antibodies
TIF1γ: transcription intermediary factor 1 gamma
SAE: small ubiquitin-like modifier activating enzyme
MDA5: melanoma differentiation-associated gene5
IFIH1: IFN induced with helicase C domain protein 1
ELISA: enzyme-linked immunosorbent assay
OR: odds ratio
CI: confidence interval
RR: relative risk
DOR: diagnostic odds ratio
AUC: area under the curve of the summary receiver operating characteristic
