# Supplementary material for: Anti-MDA5 antibody as a potential diagnostic and prognostic biomarker in patients with dermatomyositis
Source: Oncotarget. 2017 Feb 24;8(16):26552–64. doi: 10.18632/oncotarget.15716 (PMC5432278; doi:10.18632/oncotarget.15716)
Supplement: Supplementary file 1 [file oncotarget-08-26552-s001.pdf]

Anti-MDA5 antibody as a potential diagnostic and prognostic biomarker in patients with dermatomyositis

Supplementary Materials

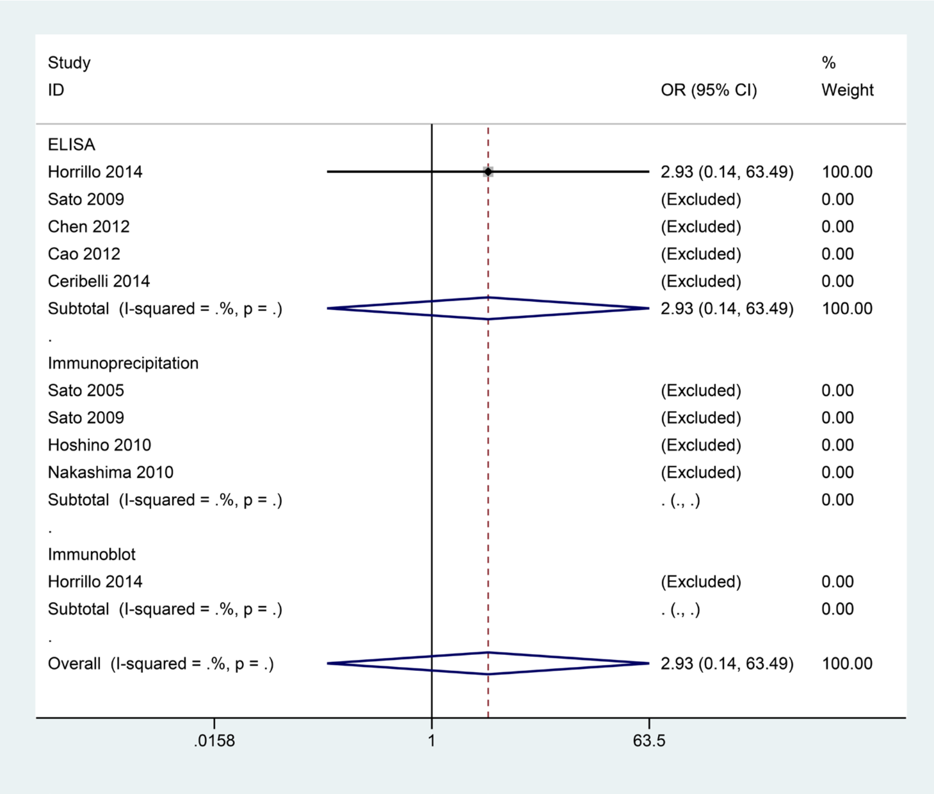

Supplementary Figure 1: Forest plot of the association between anti-MDA5 antibodies and polymyositis (PM).

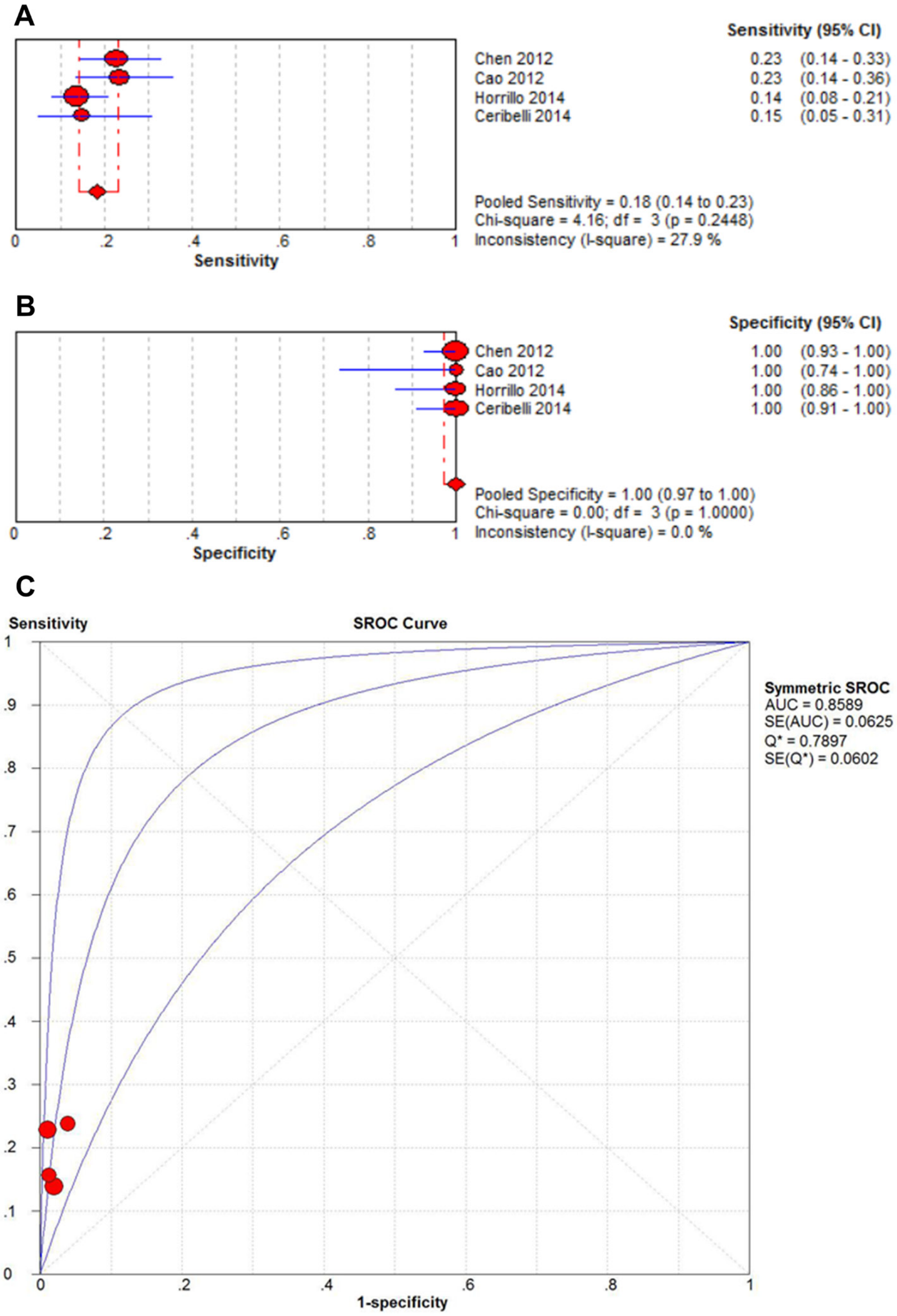

Supplementary Figure 2: Forest plots of the pooled estimates of sensitivity (**A**), specificity (**B**), and AUC (**C**) values of anti-MDA5 antibodies detected by ELISA in DM patients.

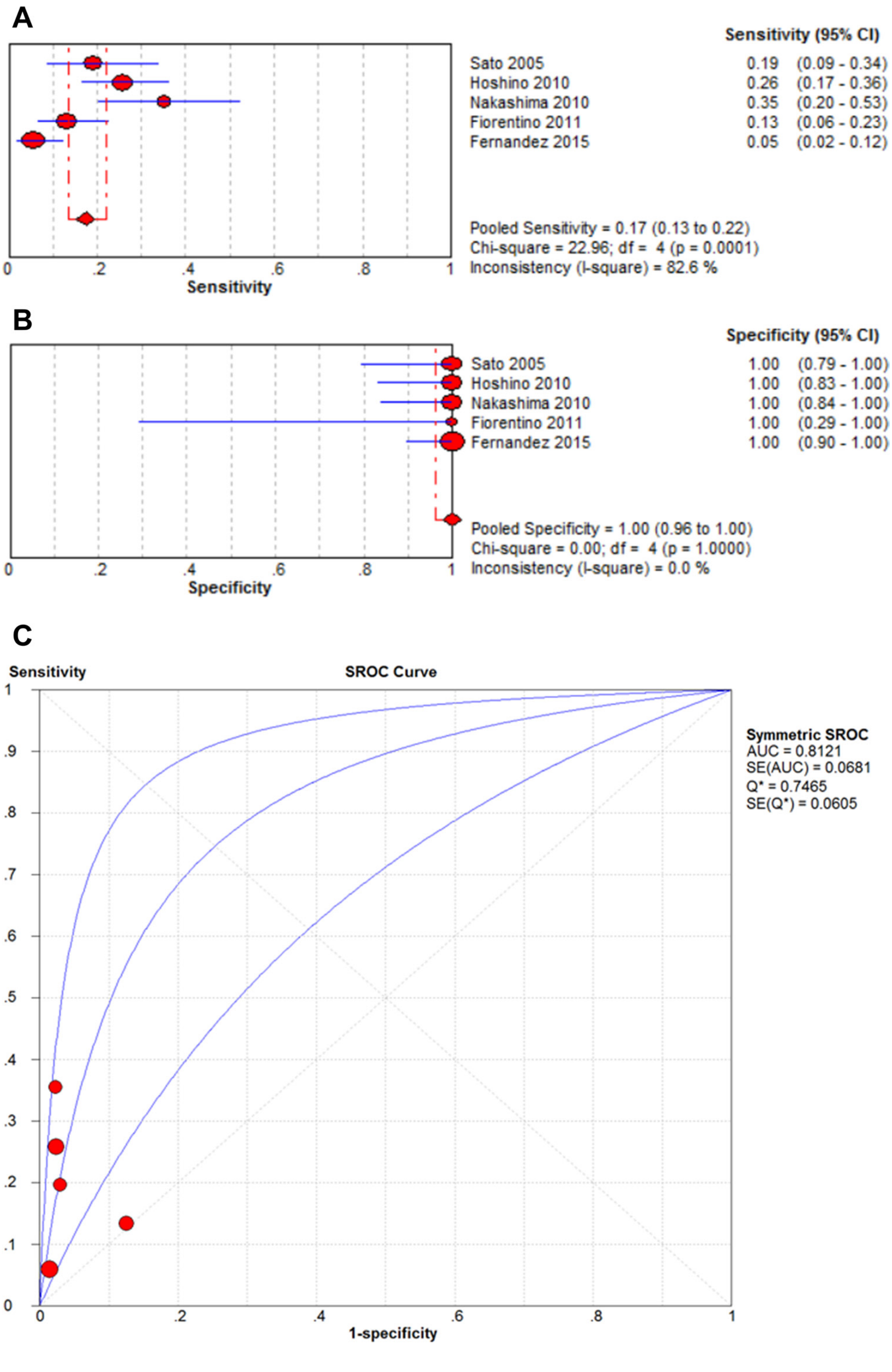

Supplementary Figure 3: Forest plots of the pooled estimates of sensitivity (**A**), specificity (**B**) and AUC (**C**) of anti-MDA5 antibodies detected by immunoprecipitation in DM patients.

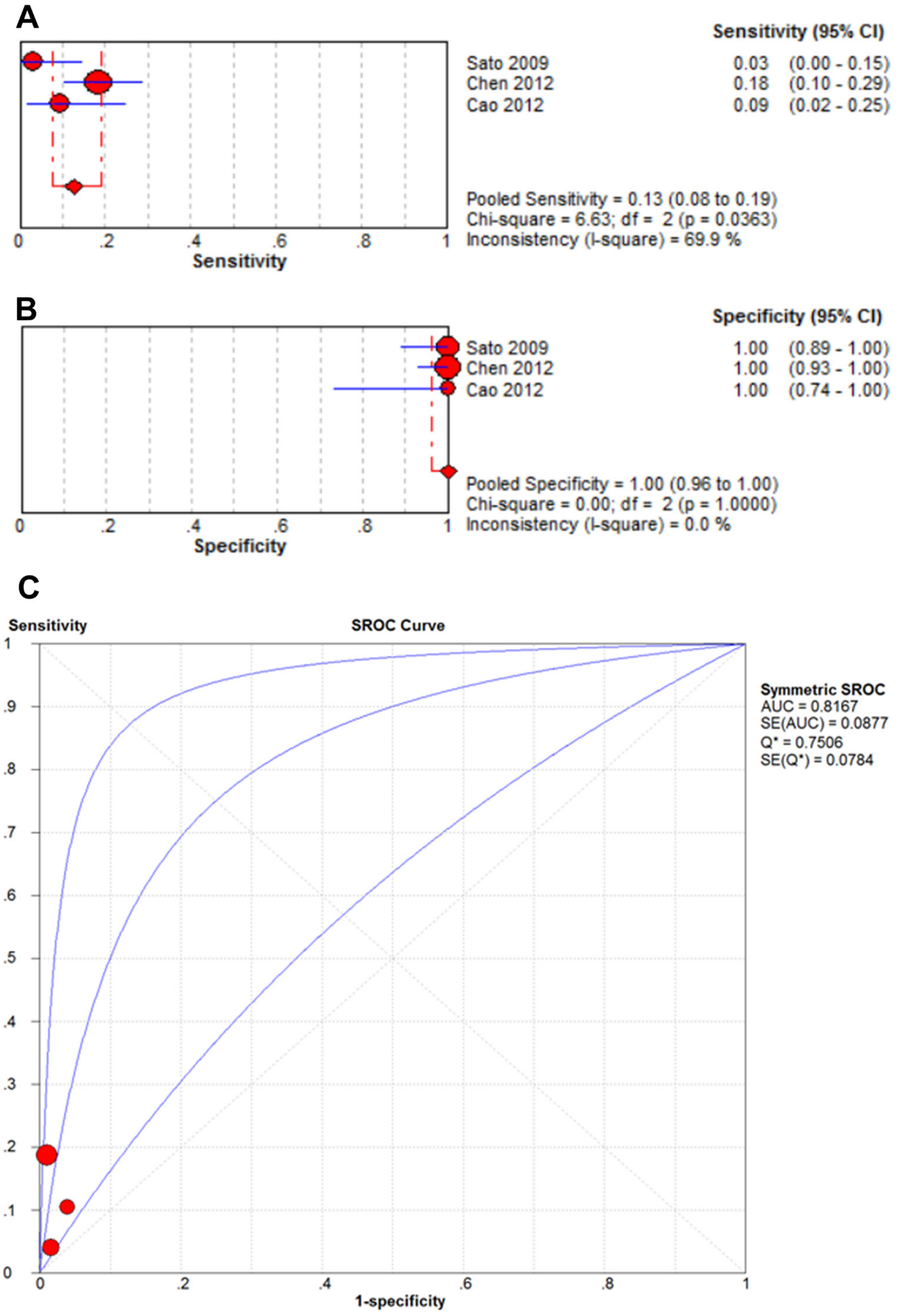

Supplementary Figure 4: Forest plots of the pooled estimates of sensitivity (**A**), specificity (**B**) and AUC (**C**) of anti-MDA5 antibodies detected by ELISA in classic DM patients.

Supplementary Table 1: Basic characteristics of the eligible studies that examined PM, DM, classic DM, and CADM cases. See Supplementary_Table_1

Supplementary Table 2: Basic characteristics of the eligible studies that examined mortality. See Supplementary_Table_2
