# Supplementary material for: Anti-MDA5 antibody as a potential diagnostic and prognostic biomarker in patients with dermatomyositis
Source: Oncotarget. 2017 Feb 24;8(16):26552–64. doi: 10.18632/oncotarget.15716 (PMC5432278; doi:10.18632/oncotarget.15716)
Supplement: Supplementary file 2 [file oncotarget-08-26552-s002.docx]

| Author and year | Disease type | Country | Ethnicity | Method | Cut-off value | Sample type | Case | | Control | |
| --- | --- | --- | --- | --- | --- | --- | --- | --- | --- | --- |
|  |  |  |  |  |  |  | A+ | Total | A+ | Total |
| Sato 2009 | PM | Japan | Japanese | ELISA using recombinant MDA5 as an antigen source | 8.0 units | sera | 0 | 53 | 0 | 32 |
| Chen 2012 | PM | China | Chinese | ELISA using recombinant MDA5 as an antigen source | 0.475 | sera | 0 | 29 | 0 | 50 |
| Cao 2012 | PM | China | Chinese | ELISA using recombinant MDA5 as an antigen source | 8.0 units/ml | sera | 0 | 15 | 0 | 12 |
| Horrillo 2014 | PM | Spain | Mediterranean | ELISA using recombinant MDA5 as an antigen source | 0.188 absorbance units | sera | 2 | 45 | 0 | 25 |
| Ceribelli 2014 | PM | Italy | European Caucasian | ELISA using recombinant MDA5 as an antigen source | NA | sera | 0 | 30 | 0 | 40 |
| Sato 2005 | PM | Japan | Japanese | immunoprecipitation using radiolabeled K562 cell extracts | NA | sera | 0 | 61 | 0 | 16 |
| Sato 2009 | PM | Japan | Japanese | immunoprecipitation using 35S-labeled HeLa cell extract | NA | sera | 0 | 53 | 0 | 32 |
| Hoshino 2010 | PM | Japan | Japanese | immunoprecipitation using biotinylated recombinant proteins | NA | sera | 0 | 6 | 0 | 20 |
| Nakashima 2010 | PM | Japan | Japanese | immunoprecipitation using [35S]methinine-labelled HeLa cell extracts | NA | sera | 0 | 47 | 0 | 21 |
| Horrillo 2014 | PM | Spain | Mediterranean | immunoblot using recombinant MDA5 | NA | sera | 0 | 45 | 0 | 25 |
| Chen 2012 | DM | China | Chinese | ELISA using recombinant MDA5 as an antigen source | 0.475 | sera | 19 | 84 | 0 | 50 |
| Cao 2012 | DM | China | Chinese | ELISA using recombinant MDA5 as an antigen source | 8.0 units/ml | sera | 15 | 64 | 0 | 12 |
| Horrillo 2014 | DM | Spain | Mediterranean | ELISA using recombinant MDA5 as an antigen source | 0.188 absorbance units | sera | 16 | 117 | 0 | 25 |
| Ceribelli 2014 | DM | Italy | European Caucasian | ELISA using recombinant MDA5 as an antigen source | NA | sera | 5 | 34 | 0 | 40 |
| Sato 2005 | DM | Japan | Japanese | immunoprecipitation using radiolabeled K562 cell extracts | NA | sera | 8 | 42 | 0 | 16 |
| Hoshino 2010 | DM | Japan | Japanese | immunoprecipitation using biotinylated recombinant proteins | NA | sera | 21 | 82 | 0 | 20 |
| Nakashima 2010 | DM | Japan | Japanese | immunoprecipitation using [35S]methinine-labelled HeLa cell extracts | NA | sera | 13 | 37 | 0 | 21 |
| Fiorentino 2011 | DM | USA | Caucasian/Latino/Pacific Islander/Asian/African American | immunoprecipitation using [35S] methionine-labeled proteins | NA | plasma | 10 | 77 | 0 | 3 |
| Fernandez 2015 | DM | USA | white or black or other | immunoprecipitation using [35S] methionine-labeled proteins | NA | sera | 5 | 91 | 0 | 34 |
| Horrillo 2014 | DM | Spain | Mediterranean | immunoblot using recombinant MDA5 | NA | sera | 14 | 117 | 0 | 25 |
| Sato 2009 | classic DM | Japan | Japanese | ELISA using recombinant MDA5 as an antigen source | 8.0 units | sera | 1 | 35 | 0 | 32 |
| Chen 2012 | classic DM | China | Chinese | ELISA using recombinant MDA5 as an antigen source | 0.475 | sera | 14 | 76 | 0 | 50 |
| Cao 2012 | classic DM | China | Chinese | ELISA using recombinant MDA5 as an antigen source | 8.0 units/ml | sera | 3 | 32 | 0 | 12 |
| Sato 2005 | classic DM | Japan | Japanese | immunoprecipitation using radiolabeled K562 cell extracts | NA | sera | 0 | 27 | 0 | 16 |
| Sato 2009 | classic DM | Japan | Japanese | immunoprecipitation using 35S-labeled HeLa cell extract | NA | sera | 2 | 35 | 0 | 32 |
| Hoshino 2010 | classic DM | Japan | Japanese | immunoprecipitation using biotinylated recombinant proteins | NA | sera | 1 | 39 | 0 | 20 |
| Nakashima 2010 | classic DM | Japan | Japanese | immunoprecipitation using [35S]methinine-labelled HeLa cell extracts | NA | sera | 2 | 22 | 0 | 21 |
| Sato 2009 | CADM | Japan | Japanese | ELISA using recombinant MDA5 as an antigen source | 8.0 units | sera | 22 | 32 | 0 | 32 |
| Chen 2012 | CADM | China | Chinese | ELISA using recombinant MDA5 as an antigen source | 0.475 | sera | 5 | 8 | 0 | 50 |
| Cao 2012 | CADM | China | Chinese | ELISA using recombinant MDA5 as an antigen source | 8.0 units/ml | sera | 12 | 32 | 0 | 12 |
| Horrillo 2014 | CADM | Spain | Mediterranean | ELISA using recombinant MDA5 as an antigen source | 0.188 absorbance units | sera | 8 | 15 | 0 | 25 |
| Xu 2016 | CADM | China | Chinese | ELISA using recombinant MDA5 as an antigen source | 0.212 absorbance units | sera | 12 | 40 | 0 | 15 |
| Sato 2005 | CADM | Japan | Japanese | immunoprecipitation using radiolabeled K562 cell extracts | NA | sera | 8 | 15 | 0 | 16 |
| Sato 2009 | CADM | Japan | Japanese | immunoprecipitation using 35S-labeled HeLa cell extract | NA | sera | 25 | 32 | 0 | 32 |
| Hoshino 2010 | CADM | Japan | Japanese | immunoprecipitation using biotinylated recombinant proteins | NA | sera | 20 | 31 | 0 | 20 |
| Nakashima 2010 | CADM | Japan | Japanese | immunoprecipitation using [35S]methinine-labelled HeLa cell extracts | NA | sera | 11 | 15 | 0 | 21 |
| Fiorentino 2011 | CADM | USA | NA | immunoprecipitation using [35S] methionine-labeled proteins | NA | plasma | 5 | 13 | 0 | 3 |
| Mateos 2014 | CADM | Spain | Spanish | immunoprecipitation using K562 cell extracts | NA | sera | 3 | 11 | 0 | 20 |
| Horrillo 2014 | CADM | Spain | Mediterranean | immunoblot using recombinant MDA5 | NA | sera | 8 | 15 | 0 | 25 |

Supplementary Table S1: Basic characteristics of the eligible studies involved PM/DM/classic DM/CADM

Note: PM = polymyositis; DM = dermatomyositis; CADM = clinically amyopathic dermatomyositis; A+ = anti-MDA5 antibody positive.
