# Supplementary material for: Anti-MDA5 antibody as a potential diagnostic and prognostic biomarker in patients with dermatomyositis
Source: Oncotarget. 2017 Feb 24;8(16):26552–64. doi: 10.18632/oncotarget.15716 (PMC5432278; doi:10.18632/oncotarget.15716)
Supplement: Supplementary file 3 [file oncotarget-08-26552-s003.docx]

Supplementary Table S2: Basic characteristics of the eligible studies involved mortality

| Author and year | Disease type | Country | Ethnicity | Method | Cut-off value | Sample type | Died from the onset of DM | A+ | | A- | |
| --- | --- | --- | --- | --- | --- | --- | --- | --- | --- | --- | --- |
|  |  |  |  |  |  |  |  | Mortality | Total | Mortality | Total |
| Nakashima 2010 | DM | Japan | Japanese | immunoprecipitation using [35S]methinine-labelled HeLa cell extracts | NA | sera | within 6 months | 6 | 13 | 2 | 24 |
| Hamaguchi 2011 | DM | Japan | Japanese | immunoprecipitation using [35S]methinine-labelled K562 cell extracts | NA | sera | within 5 years | 19 | 43 | 8 | 34 |
| Cao 2012 | DM | China | Chinese | ELISA using recombinant MDA5 as an antigen source | 8.0 units/ml | sera | within 2 months | 4 | 15 | 1 | 49 |
| Horrillo 2014 | DM | Spain | Mediterranean | immunoblot using recombinant MDA5 | NA | sera | within 2 years | 9 | 14 | 39 | 103 |
| Horai 2015 | DM | Japan | Japanese | ELISA using recombinant MDA5 as an antigen source | 8.0 units/ml | sera | NA | 3 | 10 | 0 | 20 |
| Tanizawa 2013 | DM-ILD | Japan | Japanese | immunoprecipitation using [35S]methinine-labelled HeLa cell extracts | NA | sera | NA | 13 | 20 | 2 | 20 |

Note: DM = dermatomyositis; DM-ILD = dermatomyositis with interstitial lung disease; A+ = anti-MDA5 antibody positive; A- = anti-MDA5 antibody negative.
